# MDL-800, the SIRT6 Activator, Suppresses Inflammation via the NF-*κ*B Pathway and Promotes Angiogenesis to Accelerate Cutaneous Wound Healing in Mice

**DOI:** 10.1155/2022/1619651

**Published:** 2022-04-27

**Authors:** Xiaoqi Jiang, Zhe Yao, Kangyan Wang, Lihua Lou, Kaikai Xue, Jinghao Chen, Guojian Zhang, Yu Zhang, Jiqing Du, Cai Lin, Jian Xiao

**Affiliations:** ^1^Department of Burn, Wound Repair and Regenerative Medicine Center, The First Affiliated Hospital of Wenzhou Medical University, Wenzhou 325000, China; ^2^School of Pharmaceutical Sciences, Wenzhou Medical University, Wenzhou 325035, China; ^3^Department of Orthopaedics, The Second Affiliated Hospital and Yuying Children's Hospital of Wenzhou Medical University, Wenzhou 325027, China; ^4^Department of Orthopaedics, The Affiliated Pingyang Hospital, Wenzhou Medical University, Wenzhou 325499, China; ^5^Oujiang Laboratory, Zhejiang Lab for Regenerative Medicine, Vision and Brain Health, Wenzhou 325001, China; ^6^Research Units of Clinical Translation of Cell Growth Factors and Diseases Research, Chinese Academy of Medical Sciences, Wenzhou 325027, China

## Abstract

Sirtuin 6 (SIRT6) is an NAD^+^-dependent deacetylase belonging to the sirtuin family. It has been shown to participate in wound healing and some inflammation-related disorders. However, the effect of MDL-800, a highly efficient and selective SIRT6 activator, on wound healing and inflammation has not been reported. Therefore, this study investigated whether MDL-800 confers anti-inflammatory effects and promotes wound healing and uncovered the molecular mechanisms involved. This was achieved using mouse models of full-thickness wounds. Results showed that MDL-800 significantly downregulated inflammation by attenuating the release of inflammatory mediators and improved collagen deposition and neovascularization of wounds, thereby accelerating cutaneous wound healing. Furthermore, MDL-800 significantly downregulated expression levels of TNF-*α* and IL-6 in the dorsal skin tissue of mice via the NF-*κ*B pathway. These results demonstrated that MDL-800 exerted anti-inflammatory and prohealing effects, indicating that the SIRT6/NF-*κ*B/I*κ*B signaling pathway may play an important role in wound healing.

## 1. Introduction

The skin, the largest organ in the human body, performs critical functions such as protecting internal organs from pathogen invasion and regulating body temperature [1]. Skin wounds caused by burns, trauma, and chronic have a negative impact on patients' quality of life and health and pose a significant economic and societal burden [2]. Wound healing is a complex and dynamic process that can be artificially divided into three phases: inflammatory phase, proliferation phase, and remodeling phase, all of which overlap in time and space [3, 4]. However, a complete understanding of wound healing is still lacking; especially, how to facilitate the wound healing of wounds remains a significant clinical challenge.

Neovascularization in the wound healing process occurs through a complex cascade of cellular, humoral, and molecular events. The formation of new blood vessels and the reconstruction of the vascular network are critical because they transport oxygen and nutrients to the wound site and remove metabolic waste. Local vascularization and blood circulation failure play a critical role in the delay of healing and chronic wounds. As such, promoting angiogenesis to restore vascular networks is an important therapeutic approach for cutaneous wound healing [5].

Blood clotting after injury initiates the inflammation phase, followed by the characteristic recruitment of neutrophils to the skin wound site. Neutrophils engulf and kill bacteria by producing and secreting proteases, which are responsible for necrotic tissue degradation and elimination. Subsequently, neutrophils release inflammatory mediators such as TNF-*α*, IL-1*β*, and IL-6, which boost the inflammatory response [4]. After that, macrophages phagocytose pathogens and necrotic cells and then promote cell proliferation and migration by secreting chemokines, cytokines, and growth factors [6]. Although proper inflammation is essential for protecting organisms from damage and cleaning away necrotic tissue, overactive inflammatory responses can be detrimental to wound healing [7]. Evidence shows that tight regulation of the inflammatory response, for example, shortening the inflammation period or promoting early entry into the proliferative phase is necessary for the acceleration of wound healing [8].

Nuclear factor-kappa B (NF-*κ*B), a B cell-specific transcription factor [9], plays an important role in the transcription of numerous genes involved in the initiation of the inflammatory response, thereby regulating the production of cytokines, chemokines, and growth factors in immune and acute-phase inflammatory responses [10]. As such, researchers have shown NF-*κ*B to be a critical inflammatory marker as well as a targetable pathway for treating inflammatory disorders [11]. Mounting evidence indicates that inhibiting the NF-*κ*B signaling pathway can help to speed up the healing process after an injury by regulating the inflammatory response. Therefore, it would be ideal to find a drug that can both downregulate the NF-*κ*B pathway to inhibit inflammation and promote angiogenesis to accelerate cutaneous wound healing.

Studies have shown that overexpression of sirtuin 6 (SIRT6), a member of the highly conserved sirtuin protein family can negatively modulate NF-*κ*B-dependent inflammatory responses [12, 13]. SIRT6 interacts with the NF-*κ*B p65 submit and downregulates NF-*κ*B-dependent gene expression by mediating H3K9Ac deacetylation at the molecular level. Previous evidence shows that the loss of SIRT6 in cutaneous wounds hampers diabetes-impaired wound healing by increasing the inflammatory response, decreasing angiogenesis and cells proliferation [14]. Furthermore, Hu et al. [15] found that in the corneal epithelial wound healing mouse model, SIRT6 deficiency caused delayed and incomplete wound healing. In this view, we hypothesized that MDL-800, a novel selective allosteric SIRT6 activator, could suppress NF-*κ*B signaling by activating SIRT6, which then restrains inflammation, promotes angiogenesis, and accelerates wound healing.

To test this hypothesis, we investigated the effects and potential molecular mechanism of MDL-800 in human umbilical vein endothelial cells (HUVEC) and human dermal fibroblasts (HDF) *in vitro*, as well as its protective role in mouse wound models *in vivo*. The results indicated that the MDL-800 treatment could significantly reduce inflammation and improve collagen deposition and wound neovascularization, thereby accelerating cutaneous wound healing. The findings may provide a novel insight for the wound treatment.

## 2. Materials and Methods

### 2.1. Materials and Reagents

The MDL-800 (lot number: B8384) was purchased from ApexBio Technology (United States). Dimethyl sulfoxide (DMSO) was purchased from Sigma-Aldrich (United States). Polyethylene glycol-400 (PEG-400) was purchased from SuoLaibao Technology (Beijing, China). Phosphate-buffered saline (PBS) and bovine serum albumin (BSA) were purchased from Sigma-Aldrich (MO, USA). Hematoxylin-eosin (HE) and Masson's Trichrome Stain Kit were from Beyotime Biotechnology (Shanghai, China). The primary antibodies for collagen I, collagen III, TNF-*α*, IL-6, CD 34, and *α*-SMA were purchased from Abcam (CA, UK). EdU-594 kit was purchased from Beyotime Biotechnology (Shanghai, China); 15 *μ*-slide angiogenesis was obtained from Ibidi (Martinsried, Germany); transwell chamber was purchased from Corning (NY, USA).

### 2.2. Cell Viability Assays

First, MDL-800 was dissolved in DMSO and reconfigured into 500 mM mother liquor for standby use. Human umbilical vein endothelial cells (HUVECs) and human dermal fibroblast cells (HDFs) were seeded in 96-well plates (2500 cells per well) and cultured in 10% fetal bovine serum for 24 h to determine the effect of MDL-800 on cell viability. The original medium was then replaced by fresh medium supplemented with various concentrations of MDL-800 (0, 0.5, 1, 2.5, and 5 *μ*mol/L), respectively. After 24, 48, and 72 h treatment and washing in PBS, fresh DMEM (without FBS) containing 10% CCK8 reagent was added to each well for an additional 2 h of incubation at 37°C. Finally, the absorbance at 450 nm was measured with a microplate reader to determine the viability of cells.

### 2.3. 5-Ethynyl-2′-deoxyuridine (EdU) Assay

The EdU assay was performed using an EdU-594 kit. HUVECs and HDFs were seeded in 12-well plates at target concentrations of 10^5^/mL and 5 × 10^5^/mL, respectively. After the cells adhered to the wall, fresh DMEM with different concentrations of MDL-800 (0, 0.5, and 2.5 *μ*mol/L) were added to the plate. After 24 h, the medium was changed, and the cells were cocultured with EdU working solution (1 : 1000) at 37°C for 2–4 h. The click reaction solution was then added to each well and coincubated for 30 min at room temperature in the dark. Hoechst was used to stain all cell nuclei after three washes with PBS. The images were captured with a fluorescence microscope and quantified using ImageJ.

### 2.4. Cellular Migration Assay

HUVECs in logarithmic growth were trypsinized and seeded into the upper chamber, followed by the addition of 0, 0.5, and 2.5 *μ*M MDL-800 medium (containing 10% FBS) in the lower chamber. After 6 h of incubation at 37°C, the cells were fixed for 15 min in 4% paraformaldehyde and stained for 30 min in the dark environment with 1% crystal violet. The unmigrated side was gently scraped using a wet Q-tip cotton swab to remove the cells in the upper chamber. After three PBS washes, migration activity was measured by counting cells on the lower side of the filter. Migrated cells were counted under an optical microscope.

### 2.5. Scratch Wound Assay

HUVECs and HDFs were cultured in 10% FBS/DMEM and seeded into six-well tissue culture plates. A new 200 *μ*L pipette tip was used to gently and slowly scratch a straight line across the center of each well to simulate a wound. After the scratch, the wells were rinsed three times with PBS to remove the unattached cells. The HUVECs and HDFs were then incubated at 37°C with different MDL-800 concentrations (0, 0.5, and 2.5 *μ*M). The cells were washed three times with PBS at different times (0 h, 12 h), and the gap distance was observed under the microscope and quantitatively evaluated using ImageJ.

### 2.6. *In Vitro* Tube Formation Assay

The tube formation assay on Matrigel (BD Biosciences, CA, USA) was performed to evaluate the effects of MDL-800 on HUVEC morphogenesis and tube formation capacity. After thawing at 4°C overnight, the Matrigel solution was placed in a 15-well *μ*-slide angiogenesis plate at 37°C for 1 hour to solidify. Next, the cells (1 × 10^5^ cells per well) were seeded in the 15-well *μ*-slide angiogenesis plate precoated with Matrigel and incubated at 37°C for 6 h. Also, the tube formation was examined with an inverted Nikon light microscope and quantitatively evaluated with ImageJ.

### 2.7. Western Blot Analysis

Cell protein extracts from HUVECs and HDFs treated with different MDL-800 concentrations (0, 0.5, and 2.5 *μ*M) were analyzed using Western blotting. Briefly, protein-containing samples were separated by SDS-polyacrylamide gel electrophoresis (PAGE). Proteins were transferred to PVDF membranes, blocked in 5% skim milk, and incubated with primary antibodies overnight. This was followed by incubation with the respective horseradish peroxidase-conjugated secondary antibodies for 2 h. The blots were washed three times with TBST buffer, bands were detected using an electrochemiluminescence detection system (Invitrogen, USA), and the intensity of the bands was quantified using Image Lab 3.0 software (Bio-Rad).

### 2.8. Animal Experiments

Thirty male Balb/c mice (8–12 weeks) were obtained from the Laboratory Animals Center of Wenzhou Medical University. All animals were housed in standard conditions with free access to fodder and water. All procedures complied with international ethical guidelines and the National Institutes of Health Guide for the Care and Use of Laboratory Animals; the animal permit number was wydw-2021-0102.

### 2.9. Wound Models

Thirty mice were randomly divided into three groups of ten: the saline control group, the low MDL-800 concentration group (5 mg/kg), and the high MDL-800 concentration group (25 mg/kg). Prior to mouse injection, sterile saline containing 5% polyethylene glycol 400 (PED-400) was placed in a 37°C incubator for standby use. The MDL-800 mother liquor was then diluted into different concentrations and shaken to obtain a uniform solution. The mice were anesthetized with 1% pentobarbital sodium, and their dorsal fur was shaved and sterilized with 75% ethanol. A biopsy punch was used to make two full-thickness excision wounds (8 mm in diameter) on both sides. After surgery, mice were injected daily with MDL-800 or vehicle (containing 5% PED-400) around the wound bed for 7 days. The changes in the wounds were then observed and documented. Photographs of the wounds were taken on days 0, 3, 6, 9, and 18 postoperation and analyzed using ImageJ. On days 9 and 18, mice were sacrificed after anesthesia. After that, the wound tissues were excised and fixed, paraffin-embedded, and sectioned (5 *μ*m thick).

### 2.10. Assessment of Blood Flow in the Wound Area

On days 3, 6, 9, and 18 after surgery, a laser Doppler instrument (MoorLDI-2, Moor Instruments Limited, UK) was used to assess microvascular network reconstruction in mouse back wounds. The blood flow and the results were measured using the MoorLDI Review software.

### 2.11. Hematoxylin and Eosin Staining

Tissue sections were dewaxed in xylene, rehydrated in a descending series of ethanol (100%, 90%, 80%, and 70%), and rinsed in distilled water for 5 min. Samples were counterstained with hematoxylin (5 min) and in ammonia water, for 3 min to reduce background staining. The samples were immersed in eosin for 2 min and then placed in ammonia water for 3 min. Slides were dehydrated in 70%, 80%, 90%, and 100% graded ethanol for 5 min each, then hyalinized in xylene for 15 min. Finally, the slides were sealed with neutral resin and cover-lipped for examination under a light microscope (Nikon, Tokyo, Japan).

### 2.12. Masson Trichrome Staining

Tissue sections were dewaxed in xylene, rehydrated, and stained with a Masson trichrome stain kit, as previously described. The cell nuclei were counterstained with hematoxylin (Mayer) for 3 min. Sections were washed thoroughly in distilled water and submerged in 1% acid alcohol for differentiation. Lichun red magenta solution was used to stain fibrous tissue for 2 min following the manufacturer's protocol, followed by 2 min in the phosphomolybdic acid solution for differentiation. Afterward, sections were dipped in aniline blue for 30 s, and the slides were dehydrated, sealed with neutral resin, and cover-lipped for examination under a light microscope. ImageJ software was used to determine the intensity of collagen staining.

### 2.13. Immunohistochemical Staining

Tissue sections were dewaxed in xylene for 30 min and rehydrated as previously described. The sections were then incubated at 4°C overnight with the primary antibodies of anti-collagen I (1 : 200), anti-collagen III (1 : 200), anti-IL-6 (1 : 200), or anti-TNF-*α* (1 : 200). The following day, the slides were washed three times and incubated for 2 h with secondary antibodies of goat anti-rabbit IgG or goat anti-mouse IgG (ZSGB-Bio, Beijing, China) for 2 h. DAB (ZSGB-Bio, Beijing, China) was used as a chromogen to reveal the reaction. ImageJ was used to analyze the IOD after measuring three stained sections from each group.

### 2.14. Immunofluorescence Staining

Tissue sections were incubated at 4°C overnight with rabbit CD34 mAb (1 : 200) and mouse alpha-smooth muscle actin (SMA) mAb (1 : 200). The following day, the slides were washed three times and then incubated with Alexa Fluor 488 goat anti-rabbit IgG antibody (1 : 200) and Alexa Fluor 647 goat anti-mouse IgG antibody (1 : 200). The sections were then incubated in the dark for 2 h at 37°C. Nuclei were counterstained with DAPI after 3 washes in PBS protected from light. Finally, fluorescence images were captured with a Nikon confocal laser microscope (Nikon, A1 PLUS, Tokyo, Japan) and analyzed with ImageJ software.

### 2.15. Statistical Analysis

All data are expressed as the mean ± standard error (SE). Statistical differences were performed using a one-way analysis of variance (ANOVA) followed by Tukey's test. All analyses were performed with GraphPad Prism 5 software (GraphPad Software Inc., La Jolla, CA, United States). For all tests, ^∗^*P* value < 0.05, and ^∗∗^*P* value < 0.01.

## 3. Results

### 3.1. MDL-800 Promotes Proliferation in HUVECs and HDFs *In Vitro*

**Figure 1:**
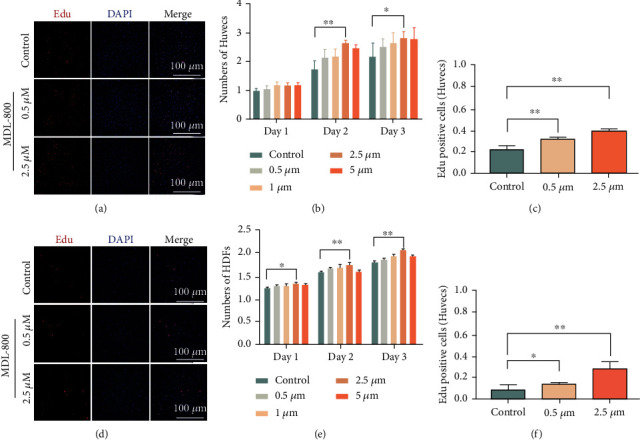
MDL-800 promotes proliferation in HUVECs and HDFs *in vitro*. (a, c) EdU staining of HUVECs and quantitative analysis of EdU-positive (RED) cell proportion. Scale bar = 100 *μ*m. *n* = 3 per group. (b) CCK8 assay of HUVECs was conducted to measure relative cell viability after being treated with different concentrations of MDL-800. (d, f) EdU staining of HDFs and quantitative analysis of EdU-positive (RED) cells proportion. Scale bar = 100 *μ*m. *n* = 3 per group. (e) CCK8 assay of HDFs was conducted to measure relative cell viability after being treated with different concentrations of MDL-800 (^∗^*P* < 0.05,  ^∗∗^*P* < 0.01).

Endothelial and fibroblast cells are essential for cutaneous wound healing because of their proliferation aids in wound closure [16]. Thus, we wondered whether MDL-800 could influence the proliferation responses of HUVECs and HDFs. HUVECs and HDFs were therefore seeded in 96-well plates and cultured with different concentrations of MDL-800. The cell numbers of the samples from different time points (24, 48, and 72 h) were then determined using CCK8 assays. The presence of 2.5 *μ*M of MDL-800 had the best effect on cell proliferation (Figures 1(b) and 1(e)). Moreover, the EdU test showed that increasing the MDL-800 dose increased the proliferation of HUVECs and HDFs (Figures 1(a) and 1(c)–1(e)), and the EdU-positive cell percentage of HUVECs and HDFs given 2.5 *μ*M MDL-800 was significantly higher than the control group; these results were consistent with the CCK-8 assay. The findings show that MDL-800 can significantly promote the proliferation of HUVECs and HDFs *in vitro*.

### 3.2. MDL-800 Promotes Migration and Tube Formation Ability of HUVECs and Promotes the Wound Healing Ability of HUVECs and HDFs *In Vitro*

**Figure 2:**
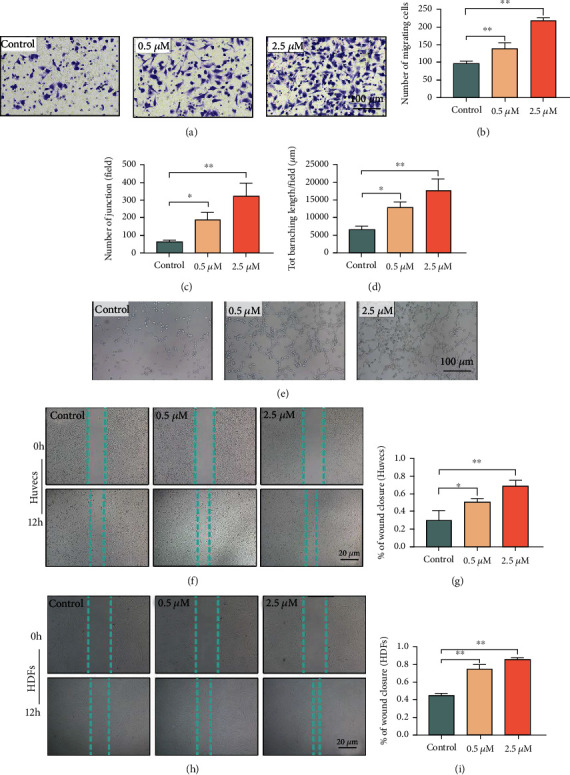
MDL-800 promoted migration and tube formation ability of HUVECs and enhanced the wound healing ability of HUVECs and HDFs. (a) Representative transwell migration assay images of HUVECS treated with different concentrations of MDL-800 (0, 0.5, and 2.5 *μ*m). Scale bar = 100 *μ*m. (b) Quantitative analysis of crystal violet stained HUVECs corresponding to transwell migration assay. *n* = 3 per group. (c, d) Quantitative analysis of junction number and tot branching length per field corresponding to tube formation assay. *n* = 3 per group. (e) Representative tube formation assay images of HUVECS treated with different concentration of MDL-800 (0, 0.5, and 2.5 *μ*m). Scale bar = 100 *μ*m. (f, g) Representative images of scratch wound healing assay of HUVECs *in vitro* at 0 and 12 h posttreatment and its quantitative analysis. Scale bar = 20 *μ*m and *n* = 3 per group. (h, i) Representative images of scratch wound healing assay of HDFs *in vitro* at 0 and 12 h posttreatment and its quantitative analysis. Scale bar = 20 *μ*m and *n* = 3 per group (^∗^*P* < 0.05,  ^∗∗^*P* < 0.01).

Cell migration from the wound edge to the wound bed is an important step in the reepithelization of skin wound healing. To see if MDL-800 can promote cell migration, we performed the transwell migration assay and used crystal violet staining to track the cell migration through the transwell membrane. The results (Figures 2(a) and 2(b)) showed that the 2.5 *μ*M MDL-800 group had the most stained HUVECs, and the HUVEC migration efficiency was proportional to MDL-800 concentration.

Meanwhile, because angiogenesis is an important process in wound healing and is responsible for transporting oxygen, nutrients, and metabolic waste, we evaluated whether MDL-800 improves the tube formation ability of HUVECs *in vitro*. In this manner, we used the tube formation assay to assess and quantify tube formation ability. Images of Matrigel tube formation are shown in Figure 2(e), demonstrating that the tube-forming ability of HUVECs was substantially increased following treatment with MDL-800. As illustrated in Figures 2(c) and 2(d), MDL-800 improved vascular network formation by increasing the number of junctions and total branching length of tubes per field; this phenomenon was positively correlated with MDL-800 concentration.

Additionally, scratch wound assays were performed to intuitively investigate the effect of MDL-800 on wound healing *in vitro*. We scratched a straight line with a new pipette tip to simulate a wound in HUVECs and HDF cells [17]. In comparison to the control group, the gap distance (Figures 2(f)–2(i)) could be narrowed down to varying degrees depending on the concentration of MDL-800. Indeed, the healing speed of scratches was faster in the high MDL-800-concentration group, which was consistent with the observed MDL-800-dependent effect in the transwell migration experiments. Thus, our findings provide evidence that the MDL-800 can promote HUVEC migration and tube formation, as well as improve the wound healing ability of HUVECs and HDFs *in vitro.*

### 3.3. MDL-800 Activates Histone H3 Deacetylation via SIRT6 in HUVECs and HDFs

**Figure 3:**
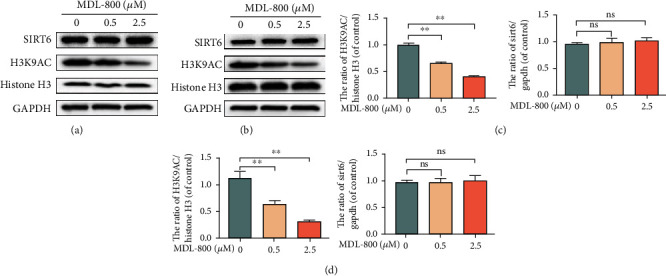
MIDL-800 activated histone H3 deacetylation of SIRT6 in HUVECs and HDFs. (a) HUVECS were treated with various concentrations of MDL-800 for 24 h and subjected to Western blot to analyze the expression of SIRT6, H3K9AC, Histone 3 and GAPDH. *n* = 3 per group. (b) HDFS were treated with various concentrations of MDL-800 for 24 h and subjected to Western blot to analyze the expression of SIRT6 H3K9AC, histone 3, and GAPDH. *n* = 3 per group (^∗^*P* < 0.05,  ^∗∗^*P* < 0.01).

SIRT6, a member of the highly conserved sirtuin family, not only modulates the deacetylation of H3K9Ac and H3K56Ac but is also involved in a variety of inflammation-related diseases [12, 13]. In this view, SIRT6 has been proposed as a promising therapeutic target in inflammation-related diseases. To see whether MDL-800 exerts its effect by activating histone H3 deacetylation of SIRT6, we looked at the expression levels of the related proteins, including H3K9AC. Western blots (Figures 3(a)–3(d)) showed that MDL-800 treatment could downregulate H3K9AC expression. Additionally, MDL-800 significantly increased histone H3 deacetylation of SIRT6 in HUVECs and HDFs; these data indicate that MDL-800 potentially inhibits the NF-*κ*B pathways to speed up wound healing.

### 3.4. MDL-800 Promotes Wound Healing *In Vivo*

**Figure 4:**
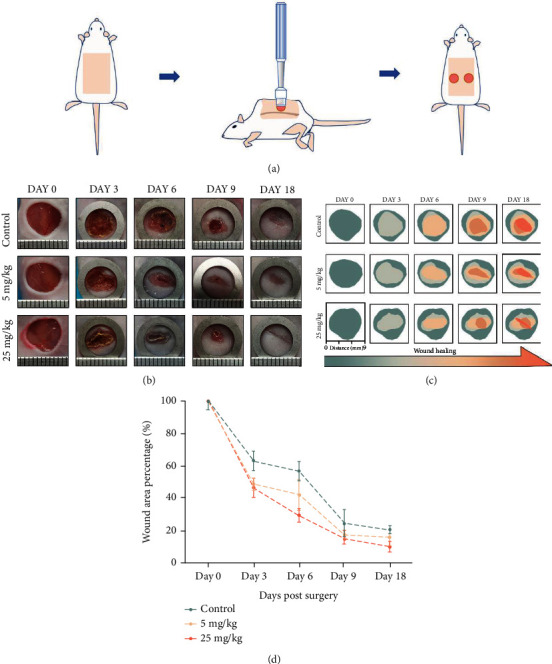
The skin wound healing promoting effects of MDL-800 *in vivo*. (a) As the animal experimental schematics show, mice were anesthetized and the dorsal fur was shaved and then two full-thickness wounds were made with biopsy punch per mice. (b) Representative images of the mice skin wound healing in the control, 5 mg/kg, and 25 mg/kg groups. The rulers in the pictures are shown in millimeters. (c) Schematic illustration of the mice skin wound healing over 18 days in control, 5 mg/kg, and 25 mg/kg group. (d) Wound area percentage at different time points (days 0, 3, 6, 9, and 18) in groups mentioned above. *n* > 3 per group (^∗^*P* < 0.05,  ^∗∗^*P* < 0.01).

Encouraged by the promising *in vitro* results, we used a full-thickness cutaneous wound model to investigate the efficacy of MDL-800 in wound healing (Figure 4(a)). We examined the effect of MDL-800 on the healing process on BALB/C mice. Briefly, 5 and 25 mg/kg MDL-800 were, respectively, applied to the full-thickness dermal wound model, with the saline group (containing 5% PED-400) serving the control group. The wounds were photographed on days 0, 3, 6, 9, and 18 postsurgery. Wounds treated with MDL-800 had a faster wound repair process (Figure 4(b)). Different colors represented the wound size of each group at days 0, 3, 6, 9, and 18.  Figure 4(c) intuitively shows a schematic diagram of the dynamic healing process. Next, we quantified the percentage of remaining unhealed wound area in each group at different time points (Figure 4(d)), and the statistical graph demonstrated that the 25 mg/kg MDL-800 treatment group (referred to as the 25 mg/kg group after) significantly increased wound healing speed when compared to other groups. The wound closure in the 25 mg/kg group was nearly complete on the 18^th^ postoperative day, indicating that MDL-800 could be a potential therapeutic candidate for wound injury management.

### 3.5. The Role of MDL-800 in Promoting Skin Tissue Development, Angiogenic Stimulation, and Collagen Deposition and Downregulating the Expression Levels of Inflammatory Factors of IL-6 and TNF-*α In Vivo*

**Figure 5:**
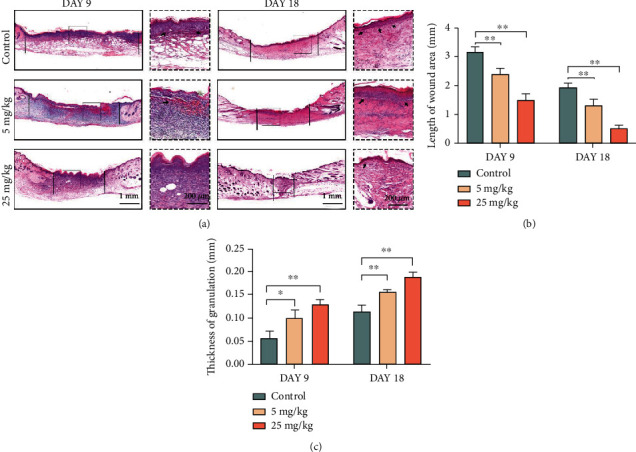
The skin tissue development promoting effects of MDL-800 *in vivo.* (a) Representative HE-stained sections of dorsal skin wound samples from different groups on day 9 and day 18. Black solid line indicated an epidermal gap. (b, c) Wound length and epidermal thickness were assessed by H&E staining images to evaluate wound healing. *n* > 3 per group (^∗^*P* < 0.05,  ^∗∗^*P* < 0.01).

Granulation tissue and the degree of reepithelialization are important indicators for assessing drug effects on skin wound repair. On days 9 and 18, tissue slices from each group were stained with hematoxylin-eosin to examine the granulation tissue regeneration (Figures 5(a) and 5(b)). In line with the previous findings, the length of the remaining unhealed wound area was shorter in the 5 mg/kg MDL-800 group than in the control group, and the 25 mg/kg group also had the shortest wound length and fastest healing speed. Furthermore, in the HE staining assay, the 25 mg/kg group had the thickest central epidermis of the wound (Figure 5(c)). The regenerated tissue in the wound center of the control group was thinner than that of the 5 mg/kg group, whereas wounds in the 25 mg/kg group were thicker and flatter with normal epithelization and less inflammatory cell infiltrations, as evidenced by HE staining.

**Figure 6:**
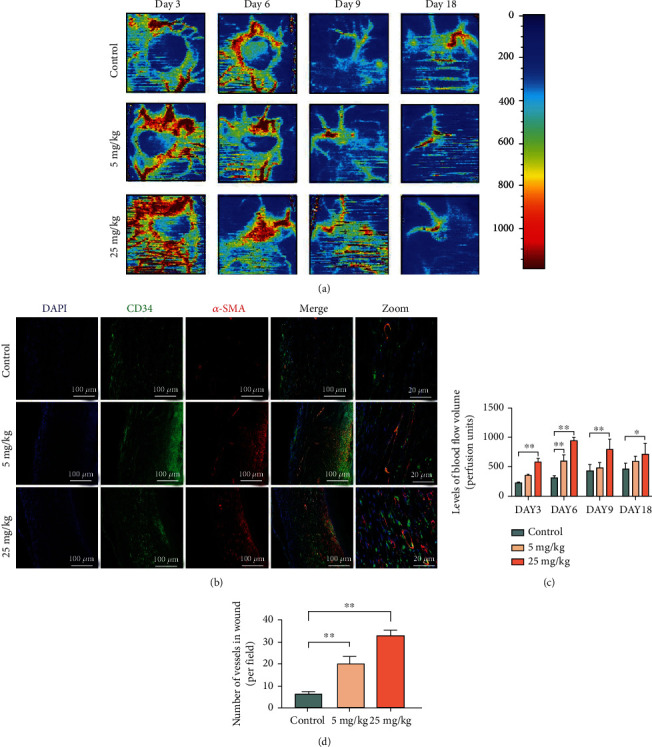
The angiogenic stimulation ability of MDL-800 *in vivo*. (a) Representative color laser Doppler images are taken to value subcutaneous blood flow on postsurgery days 3, 6, 9, and 18. (b) Coimmunofluorescence staining of CD-31 (green) and alpha-smooth muscle actin (red) of the control, 5 mg/kg, and 25 mg/kg groups on day 9. Scale bar = 100 *μ*m or 20 *μ*m. (c) Quantitative data of blood flow signal intensity of cutaneous wounds of the groups mentioned above. *n* = 3 per group. (d) Quantification of newly formed vessels per field corresponding to immunofluorescence staining of CD-31 and *α*-SMA. *n* = 3 per group (^∗^*P* < 0.05,  ^∗∗^*P* < 0.01).

Furthermore, we investigated the effects of MDL-800 *in vivo* based on its strong angiogenic stimulation ability in HUVECs. Hence, the blood flow at the wound site and newly formed vascular networks were investigated. The microvascular network reconstruction in the wound area was then visualized using LDBF, blood flow indicator: the redder the color, the greater the blood flow. The blood flow intensity in the low MDL-800-concentration group was significantly higher than that in the control group (Figures 6(a) and 6(c)), which was consistent with the *in vitro* experiments. Similarly, MDL-800 concentration influenced blood flow at the wound bed.

Because wound healing necessitates angiogenesis, we used immunostaining for *α*-SMA and CD31 as vasculature markers to investigate wound angiogenesis. Strong positive staining of *α*-SMA, CD31, and DAPI on the wound bed, as shown in Figures 6(b) and 6(d), suggested that MDL-800 treatment significantly increased the number of neovascularization, implying that MDL-800 boosted angiogenesis and promoted vascular network reconstruction and wound healing. Quantitative assessment of blood vessel density within the wound sites showed that the 25 mg/kg group had more and denser neovascularization at the wound site compared to the 5 mg/kg group and the control group.

**Figure 7:**
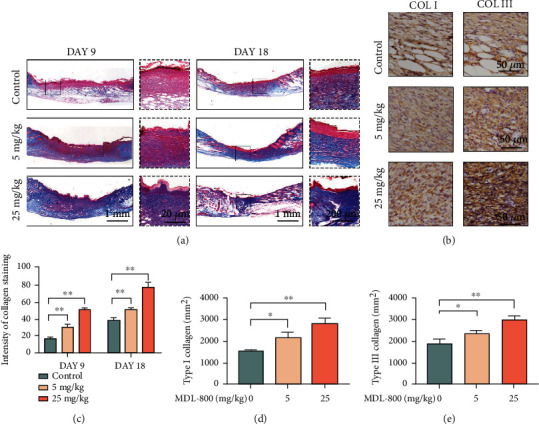
The capacity of promoting collagen deposition of MDL-800 *in vivo*. (a) Representative Masson's trichrome-stained sections of dorsal skin wound samples from different groups on day 9 and day 18. (b) Immunostaining for type I collagen and type Ill collagen of the control, 5 mg/kg, and 25 mg/kg groups on day 9. Scale bar = 50 *μ*m. (c) Quantification analysis of relative collagen density corresponding to Masson's trichrome staining of the groups mentioned above on day 9 and day 18. *n* > 3 per group. (d, e) Quantification analysis of relative density of type I collagen and type Ill collagen of the groups mentioned above on day 9. *n* > 3 per group (^∗^*P* < 0.05,  ^∗∗^*P* < 0.01).

Appropriate collagen deposition and remodeling are important factors in wound healing in the later stages. On days 9 and 18, we measured the collagen content using Masson trichrome staining and immunostaining of COL I and COL III (Figures 7(a) and 7(c)). The pale blue collagen was found in both the control and the low MDL-800-concentration groups. Importantly, the compact and orderly collagen arrangement, as well as mature skin appendages, were observed in the high MDL-800-concentration group (use black arrows on the diagram). Notably, immunostaining data showed that intracutaneous injection of 5 mg/kg MDL-800 increased the mean optical density of COL I and COL III in the dorsal skin compared to the control group, while the improvement of mean optical density of COL I and COL III was significantly greater in the 25 mg/kg group. These findings demonstrated that MDL-800 increased collagen tissue deposition and granulation tissue formation during the wound healing process.

**Figure 8:**
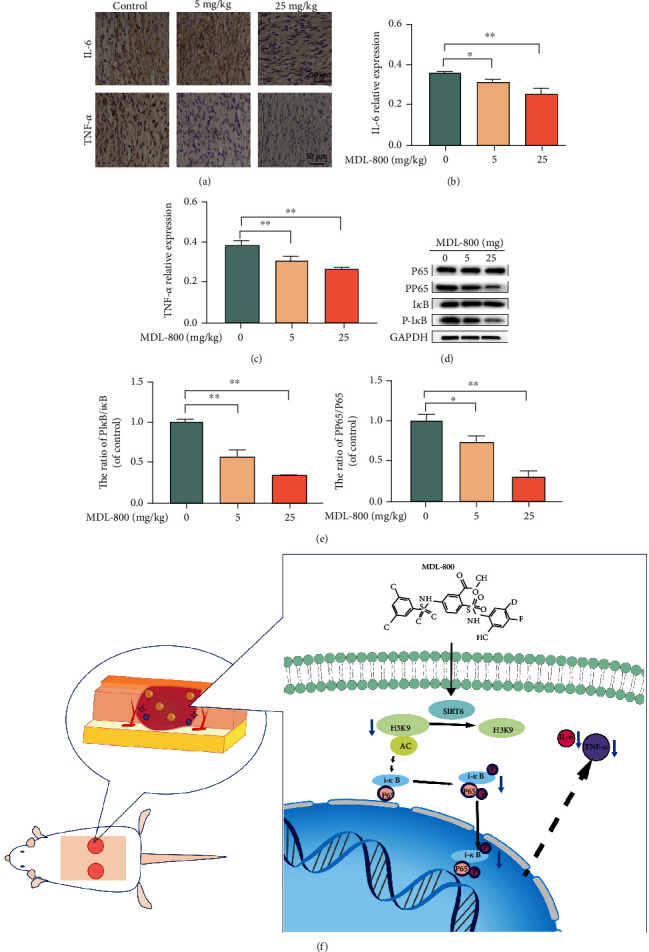
MDL-800 inhibited the NF-KB pathway in cutaneous wound. (a) Immunohistochemistry staining images of IL-6 and TNF-*α* of the control, 5 mg/kg, and 25 mg/kg groups on day 9 postsurgery. (b, c) Quantification of relative density of IL-6 and TNF-*α* of the groups mentioned above on day 9 postsurgery. *n* = 3 per group. (d, e) Mice treated with various concentrations of MDL-800 and subjected to Western blot to analyze the expression of P65, PP65, l*κ*B, P-l*κ*B, and GAPDH. *n* = 3 per group. (f) Schematic diagram of Mdl-800-mediated regulation of anti-inflammation and angiogenesis by inhibiting NF-*κ*B/P65 pathway to accelerate wound healing (^∗^*P* < 0.05,  ^∗∗^*P* < 0.01).

Canonical proinflammatory cytokines IL-6 and TNF-*α* are linked to inflammatory reactions in the early stages of wound healing [18, 19]. The expression of IL-6 and TNF-*α* was then determined by tissue immunostaining. As shown in Figures 8(a)–8(c), the control group exhibited a more severe inflammatory response and higher expression of IL-6 and TNF-*α*, the 5 mg/kg MDL-800 group had less inflammatory effect, and the 25 mg/kg group exhibited the lowest IL-6 and TNF-*α* expression. These findings demonstrated that MDL-800 suppressed inflammation and reduced the expression levels of inflammatory factors IL-6 and TNF-*α* within the wound site.

### 3.6. MDL-800 Inhibits the NF-*κ*B Pathway in the Cutaneous Wounds

We sought to investigate the underlying mechanisms with in-depth resolution. The Western blot results showed higher expression of NF-*κ*B pp65 and pI*κ*B in the 0.9% saline group compared to the 5 mg/kg and 25 mg/kg groups (Figures 8(d) and 8(e)). MDL-800 treatment significantly inhibited the phosphorylation of NF-*κ*B p65 and I*κ*B*α* in mouse skin wounds, thus lowering the expression of inflammatory factors IL-6 and TNF-*α*. As a result, the following molecular mechanisms were proposed: MDL-800 may regulate H3K9Ac and subsequently suppression of the NF-*κ*B pathway, inhibit the inflammatory response, and promote wound healing.

## 4. Discussion

The wound healing process occurs in different stages, beginning with rehemostasis and progressing through inflammation, proliferation, and maturation phases [20]. Inflammation is required during the inflammatory phase for the invasion of environmental pollutants and the removal of tissue debris. However, excessive inflammation may cause damage to normal tissues, for instance delaying wound healing or leading to fibrosis-related diseases [21]. Appropriate regulation of the inflammatory response facilitates the shortening of the inflammation period and entry into the proliferative phase, which is critical for accelerating wound healing [22].

In this study, MDL-800 could rapidly and significantly reduce IL-6 and TNF-*α* expression in the wound at 9 days postoperation, indicating the ability of MDL-800 to downregulate inflammation. The wounds in the high-dose MDL-800 treatment group appeared to have reepithelialized better, as evidenced by HE-stained sections (Figure 5(a)). At 9 days postwounding, the 25 mg/kg MDL-800 group had the most compact and orderly collagen arrangement accompanying with mature skin appendages. TNF-*α* downregulation is likely to be responsible for such performance because the inflammatory cytokines TNF-*α* can increase matrix metalloproteinase (MMP) secretion and collagen degradation and suppress collagen I/III synthesis [23].

Angiogenesis in the newly generated dermis is required for proper wound repair because neovascularization can provide the tissue with nutrients and oxygen [5, 24]. Angiogenesis plays a key role in the healing of cutaneous wounds because it promotes cell proliferation, migration, and other metabolic activities [25, 26]. The color ultrasonic Doppler images of wound tissues displayed in Figure 7(a) provide a visual representation of angiogenesis at the wound site. Blood flow in the wounds was also higher in the high-dose group when compared to the control and low-dose groups. In addition, accompanied by numerous *α*-SMA-CD31-stained blood vessels (Figure 7(b)), MDL-800 significantly increased blood flow in the regenerated tissue during the wound healing process, indicating that MDL-800 can not only promote the formation of blood vessels but also accelerate the maturation of functional blood vessels as blood flows *in vivo*.

Emerging evidence indicates that sirtuin 6 potentially plays a role in wound healing, including skin and corneal wound healing [14, 15]. Sirt6 deficiency delays cutaneous wound healing in diabetic mice, resulting in delayed and incomplete corneal wound healing. Intriguingly, SIRT6 overexpression reduces the expression of the proinflammatory transcription factor NF-*κ*B [27]. Overall, the findings indicate that SIRT6 functions as a negative regulator of vascular inflammatory responses. As such, activating SIRT6 may be beneficial in endothelial dysfunction-related inflammation diseases. At the molecular level, SIRT6 deacetylates histone H3 on acetylated K9, K18, and K56 (H3K9Ac, H3K18Ac, and H3K56Ac), resulting in transcriptional suppression of specific genes [28–30]. In the present work, we discovered that MDL-800, a known allosteric activator of SIRT6 [31], could induce SIRT6-mediated deacetylation of histone H3 in HUVEC and HDF cells. It is worth noting that histone H3 deacetylation is positively correlated with MDL-800 concentration. Importantly, we found that H3K9Ac levels are associated with accelerated wound healing and downregulated inflammatory cytokines. These findings collectively shed light on the potential role of MDL-800 in wound healing by suppressing NF-*κ*B signaling.

Moreover, we looked into a possible link between the anti-inflammatory effect of MDL-800 on Sirt6 and NF-*κ*B signaling. We found that MDL-800 could significantly suppress phosphorylation of NF-*κ*B p65 and I*κ*B via the axis of SIRT6 and H3K9, which assisted in the downregulation of inflammatory response and wound healing acceleration. However, the effects of MDL-800 on promoting refractory wound healing such as burn wounds, diabetic wounds, and infected wounds are still unknown; further research into these possibilities may be required in the future.

## 5. Conclusion

MDL-800, a novel SIRT6 activator, promotes endothelial cell proliferation, migration, tube formation, and fibroblast proliferation. As a result, angiogenesis and migration toward the wound center are accelerated, resulting in faster wound healing. Meanwhile, MDL-800 suppresses the inflammatory response and downregulates the expression of inflammatory factors IL-6 and TNF-*α* via the NF-*κ*B pathway, which aids in wound healing. These findings suggest that MDL-800 may provide a potent and efficient therapy for cutaneous wounds, implying the potential of MDL-800 in the treatment of wound patients.

## Data Availability

All data supporting the conclusions of this manuscript are provided in the text and figures. Please contact the authors for data requests.
